# Comprehensive genetic profiling and molecularly guided treatment for patients with primary CNS tumors

**DOI:** 10.1038/s41698-024-00674-y

**Published:** 2024-08-14

**Authors:** Julia C. Kuehn, Patrick Metzger, Nicolas Neidert, Uta Matysiak, Linda Gräßel, Ulrike Philipp, Sabine Bleul, Thomas Pauli, Julia Falkenstein, Henriette Bertemes, Stepan Cysar, Maria Elena Hess, Anna Verena Frey, Jesús Duque-Afonso, Elisabeth Schorb, Marcia Machein, Jürgen Beck, Oliver Schnell, Nikolas von Bubnoff, Anna L. Illert, Christoph Peters, Tilman Brummer, Marco Prinz, Cornelius Miething, Heiko Becker, Silke Lassmann, Martin Werner, Melanie Börries, Justus Duyster, Dieter H. Heiland, Roman Sankowski, Florian Scherer

**Affiliations:** 1 Department of Medicine I, Medical Center—University of Freiburg, Faculty of Medicine, University of Freiburg, Freiburg, Germany; 2 Comprehensive Cancer Center Freiburg, Medical Center—University of Freiburg, Faculty of Medicine, University of Freiburg, Freiburg, Germany; 3 Institute of Medical Bioinformatics and Systems Medicine, Medical Center—University of Freiburg, Faculty of Medicine, University of Freiburg, Freiburg, Germany; 4 German Cancer Research Center (DKFZ), Heidelberg, Germany; 5 Department of Neurosurgery, Medical Center—University of Freiburg, Faculty of Medicine Freiburg, University of Freiburg, Freiburg, Germany; 6 German Cancer Consortium (DKTK Partner site Freiburg, a partnership between DKFZ and Medical Center—University of Freiburg, Heidelberg, Germany; 7 Institute for Surgical Pathology, Medical Center—University of Freiburg, Faculty of Medicine, University of Freiburg, Freiburg, Germany; 8 Faculty of Biology, University of Freiburg, Freiburg, Germany; 9 Department of Neurosurgery, Universitätsklinikum Erlangen, Friedrich-Alexander University (FAU) Erlangen-Nürnberg, Erlangen, Germany; 10 Department of Hematology and Oncology, University Hospital Schleswig-Holstein, Luebeck, Germany; 11 Department of Medicine III, Faculty of Medicine, Klinikum Rechts der Isar, Technical University Munich (TUM), Munich, Germany; 12 Signalling Research Centres BIOSS and CIBSS, University of Freiburg, Freiburg, Germany; 13 Institute of Molecular Medicine and Cell Research, ZBMZ, Faculty of Medicine, University of Freiburg, Freiburg, Germany; 14 Institute of Neuropathology, Medical Center—University of Freiburg, Faculty of Medicine, University of Freiburg, Freiburg, Germany

**Keywords:** CNS cancer, CNS cancer, Molecular medicine, Diagnostic markers

## Abstract

Despite major advances in molecular profiling and classification of primary brain tumors, personalized treatment remains limited for most patients. Here, we explored the feasibility of individual molecular profiling and the efficacy of biomarker-guided therapy for adult patients with primary brain cancers in the real-world setting within the molecular tumor board Freiburg, Germany. We analyzed genetic profiles, personalized treatment recommendations, and clinical outcomes of 102 patients with 21 brain tumor types. Alterations in the cell cycle, BRAF, and mTOR pathways most frequently led to personalized treatment recommendations. Molecularly informed therapies were recommended in 71% and implemented in 32% of patients with completed molecular diagnostics. The disease control rate following targeted treatment was 50% and the overall response rate was 30%, with a progression-free survival 2/1 ratio of at least 1.3 in 31% of patients. This study highlights the efficacy of molecularly guided treatment and the need for biomarker-stratified trials in brain cancers.

## Introduction

Biomarker-guided therapies have revolutionized clinical management of patients with cancers^1–3^. In addition to significant advances in drug development, innovative next-generation sequencing (NGS) technologies facilitate the identification of patients with targetable genetic aberrations in real-time and enable novel molecularly stratified clinical trials^4–6^. This major technical progress has substantially enhanced the genetic characterization and classification of central nervous system (CNS) tumors, which are a highly heterogeneous group of cancer types and encompass more than 120 different entities^7^. However, in contrast to other solid cancers such as non-small cell lung cancer or melanoma and despite extensive preclinical research efforts, targeted and biomarker-informed treatment options are highly limited for CNS tumors, and current systemic therapeutic strategies still largely rely on conventional chemotherapy^8–11^. The phase III INDIGO trial has demonstrated the efficacy of the dual IDH1/2 inhibitor vorasidenib in *IDH*-mutant gliomas, which led to a priority review by the FDA and highlights the need for molecular profiling and precision medicine in this field^12^. Yet, most clinical trials that assess biomarker-stratified therapies are still not accessible for patients with brain tumors and furthermore, a large proportion of targeted drugs do not achieve sufficient concentrations in the CNS compartment due to the blood-brain barrier. Thus, many therapies that have been successfully tested in extracerebral malignancies may not be effective in patients with brain cancers, despite the fact that these tumors share similar alterations^13,14^.

Molecular tumor boards (MTB) might present a viable avenue for biomarker-guided and personalized treatment outside clinical trials for brain cancer patients experiencing disease progression following conventional therapies^15–17^. The MTB of the Comprehensive Cancer Center Freiburg (MTB-FR), Germany, has established standardized workflows for comprehensive entity-specific molecular diagnostics of tumor tissue, interdisciplinary evaluation and analysis of genomic profiles along with conventional clinical parameters, and biomarker-informed treatment recommendations in cancer patients enrolled in a prospective observational study (DRKS00025847)^15^.

Here, we report the results of individual molecular testing and clinical outcomes following personalized treatment recommendations of 102 patients with 21 different primary brain tumor entities enrolled in the MTB-FR prospective observational study between 2018 and 2023.

## Results

### Study cohort and patient characteristics

**Fig. 1:**
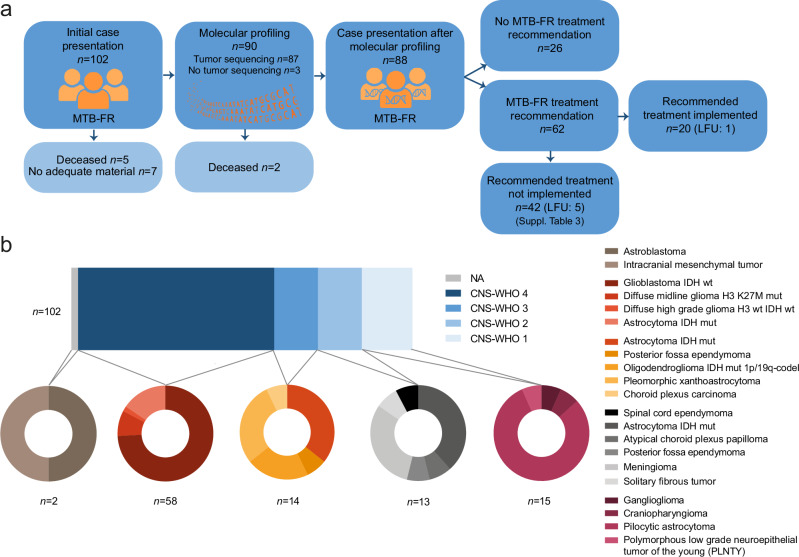
Workflow and distribution of entities in the MTB-FR primary CNS tumor cohort. **a** Schematic overview of the MTB-FR workflow and numbers of primary CNS tumor patients assessed during this workflow. LFU, Loss to follow-up. **b** Distribution of brain cancer entities based on CNS-WHO grades defined by the WHO 2021 classification (*n* = 102). wt wildtype, mut mutated, codel co-deleted.

Between September 2018 and October 2023, 102 patients with primary brain tumors were included in the MTB-FR observational study and assessed for this analysis (Fig. 1a and Supplementary Fig. 1a). Tumor molecular profiling was successfully performed and subsequently discussed in a multidisciplinary fashion within the MTB-FR in 87 patients (85%), while tumor material was insufficient for molecular analyses in 7 patients, 7 patients died before or during molecular testing and in one case, tumor sequencing could not be successfully completed (Fig. 1a). Biomarker-informed therapies were recommended for 62 patients (71%) and 20 patients (32%) ultimately received the recommended targeted treatment after subsequent disease progression and drug approval by the health insurance (Fig. 1a). In 42 patients, the recommended therapy has not been implemented either due to poor performance status following cancer progression and decision for best supportive care, loss to follow-up (LFU), rejection of drug coverage by the health insurance, or unexpected prolonged response to standard therapy (Fig. 1a and Supplementary Data 1). The median follow-up after enrollment in the MTB-FR was 829 days (range: 220–2131 days).

Table 1 provides an overview of the clinical characteristics of all patients included in this study. At the initial case presentation in the MTB-FR, the median age of patients was 46.5 years (range: 20–81), with a median Karnofsky Performance Status (KPS) of 90% (range: 50%–100%, Table 1 and Supplementary Fig. 1b, c). Patients received in median 2 prior treatment lines before enrollment (range: 0–8) (Table 1 and Supplementary Fig. 1d). Our cohort was highly heterogeneous with 21 different brain tumor entities, spanning all CNS-WHO grades from 1 to 4 (Fig. 1b). The majority of cases were CNS-WHO grade 4 tumors (57%), with glioblastoma representing the most common cancer type (43 patients, 42%), followed by CNS-WHO grade 1 tumors (15 patients, 15%), CNS-WHO grade 3 tumors (14 patients, 14%), and CNS-WHO grade 2 tumors (13 patients, 13%). Patients were enrolled in the MTB-FR observational study in median 16 months (range: 0.6–399 months) after diagnosis, with CNS-WHO grade 4 patients being included in median 9.5 months, CNS-WHO grade 3 patients 69.4 months, CNS-WHO Grade 2 patients 48.8 months, and CNS-WHO Grade 1 patients 27.6 months after initial identification of the tumor (Supplementary Fig. 1e). Median time from the initial case presentation to treatment recommendation was 80 days.

**Table 1:** Clinical characteristics of the MTB-FR primary brain tumor cohort

Clinical characteristics	Cohort *n* = 102
Age in years, median (range)	46.5 (20–81)
Sex, male:female ratio	1.9 (67:35)
WHO Grade, no. (%)	
4	58 (57)
3	14 (14)
2	13 (13)
1	15 (15)
NA	2 (2)
Year of enrollment, no. (%)	
2018	2 (2)
2019	9 (9)
2020	10 (10)
2021	30 (29)
2022	27 (26)
2023	24 (24)
Treatment lines before enrollment, median (range)	2 (0–8)
KPS at enrollment, median (range)	90 (50–100)
Time initial diagnosis to enrollment in days, median (range)	486.5 (19–12133)

*KPS* Karnofsky performance status, *no*. number.

### Molecular profiling

Tumor molecular profiling was performed in 90 patients (88%) either from FFPE tissue (97%) or from fresh frozen specimens (3%, Supplementary Fig. 2a). In 56% of cases, tumor samples from the initial diagnosis were profiled; tissue from relapse or progression time points was used in 44%. Tumor specimens were either obtained through resection (81%) or stereotactic biopsies (19%) of tumor lesions in the brain (Supplementary Fig. 2a and Supplementary Data 2). The median tumor cell content was 70%, with a range of 10–95% (Supplementary Fig. 2b and Supplementary Data 2).

We performed whole exome sequencing (WES) including germline analyses in 12 cases (13%), while tumors from 75 patients (83%) were genotyped by targeted NGS assays applying either large sequencing panels spanning at least 500 genes (57 patients, 63%) or smaller panels/qPCR methods that cover less than 500 genes (18 patients, 20%) (Supplementary Fig. 2c, Methods). In addition, immunohistochemistry beyond standard diagnostic procedures covering Programmed cell death ligand-1 (PD-L1), HER2, Somatostatin receptor, PTEN, and phosphorylated ERK were conducted in the majority of cases either to identify additional targets or to validate sequencing results. For a subset of patients, methylome analyses (29%) and RNA sequencing (14%) were added to the diagnostic portfolio (Supplementary Fig. 2c).

**Fig. 2:**
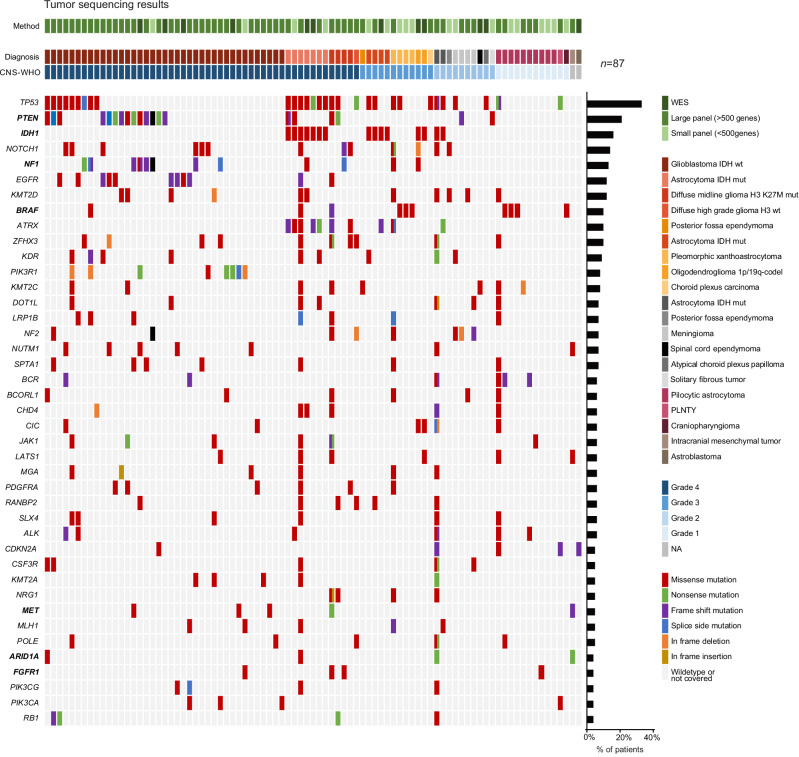
Tumor sequencing results. Case-level mutational profiles of 87 primary brain tumors genotyped by WES or targeted next-generation sequencing using capture panels. Each column represents a tumor sample, each row represents a gene. Genes with at least four recurrent mutations in the cohort are shown. The map was manually clustered to highlight mutation co-occurrence. The percentage of patients carrying an alteration in each gene is shown as a bar graph on the right. Sequencing method, diagnosis as well as CNS-WHO grades are depicted as a color code in the first three rows. Bold gene names indicate genetic alterations resulting in treatment recommendations. WES Whole exome sequencing, wt wildtype, mut mutated, codel co-deleted, PLNTY polymorphous low-grade neuroepithelial tumor of the young.

Tumor sequencing results from different technologies were presented and discussed for 87 cases (Fig. 1a). Molecular profiles including single nucleotide variants (SNVs), copy number variants (CNVs), gene fusions, tumor mutational burden (TMB) and PD-L1-Scores (CPS/TPS) are depicted in Fig. 2 and Supplementary Fig. 3 and listed in Supplementary Data 3. The most commonly detected variants were found in *TP53* (*n* = 30), *PTEN* (*n* = 20), *IDH1* (*n* = 15), *NOTCH1* (*n* = 13), *NF1* (*n* = 12), *EGFR* (*n* = 11), *KMT2D* (*n* = 11), *ATRX* (*n* = 10), and *BRAF* (*n* = 10) genes, largely consistent with previously reported mutational landscapes in primary brain tumors^18,19^. *BRAF* fusions with *KIAA1549* as a partner gene were observed in 3 patients, all of which were diagnosed with pilocytic astrocytomas. Notably, we detected two *NTRK* fusions, one found in a patient with glioblastoma, and one in a pilocytic astrocytoma (Supplementary Fig. 3). Furthermore, one-third of CNS-WHO grade 4 tumors (*n* = 19) were found to harbor *EGFR* amplifications, while *FGFR* gains or *PTEN* losses were detected in four patients, respectively (Supplementary Fig. 3). TMB was available in a subset of patients but no recommendations were based on this parameter, as its predictive value remains unclear in brain cancer entities^20^.

In four cases, our extended molecular profiling resulted in a revision of the initial diagnosis. In one case, the detection of an *IDH1* D375N mutation led to re-classification from giant cell glioblastoma to astrocytoma CNS-WHO grade 4. In a separate patient, the diagnosis was changed from glioblastoma to pleomorphic xanthoastrocytoma due to the CNV profile. In two cases, the identification of entity-specific fusions suggested a re-classification of a meningioma CNS-WHO grade 2 to intracranial mesenchymal tumor (*ATF1::ESWR1* fusion) and glioblastoma to a polymorphous low-grade neuroepithelial tumor of the young (PLNTY, *FGFR2::CTNNA3* fusion).

### Molecularly guided treatment recommendations and clinical outcomes

**Fig. 3:**
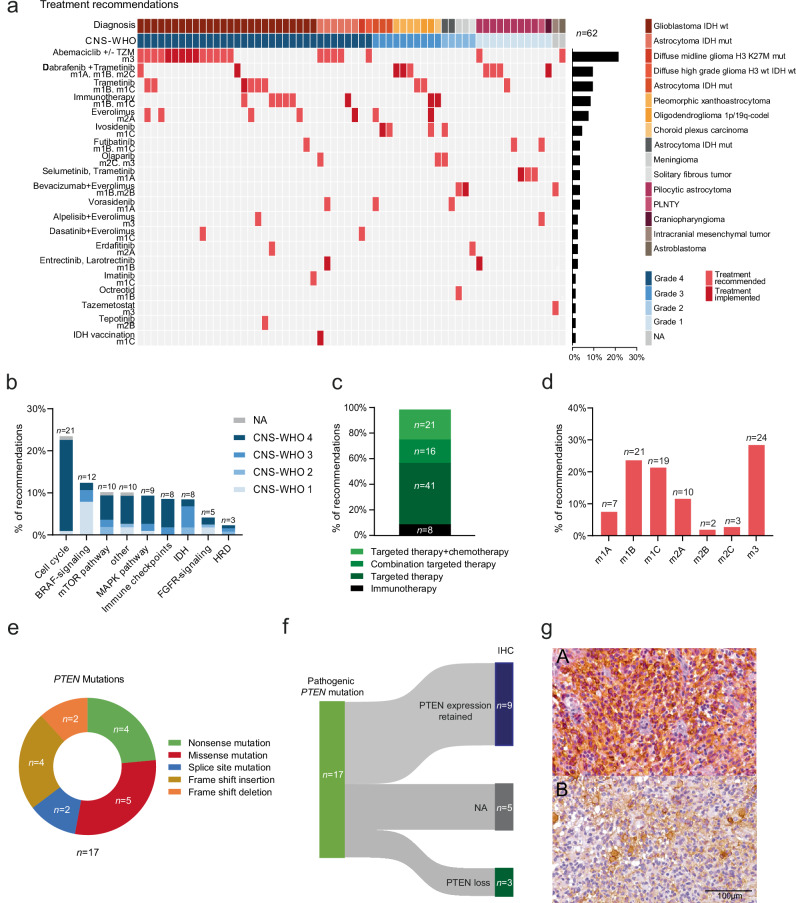
MTB-FR treatment recommendations. **a** Case-level profile of MTB-FR therapy recommendations for 62 patients. Each column represents one case. Each row represents one recommendation. The recommended agent is stated for each row along with the respective NCT evidence levels. The map was manually clustered based on the cancer entity and recommended treatment. The percentage of recommendations is shown as a bar graph on the right. Implemented recommendations are shown in dark red rectangles, recommended treatment that was not implemented is shown in light red rectangles. Diagnosis and CNS-WHO grades are color-coded in the first rows. Wt wildtype, mut mutated, codel co-deleted, PLNTY polymorphous low-grade neuroepithelial tumor of the young, TZM Temozolomide. **b** Recommendations are assigned to 9 biological processes. The proportion of these recommendations to the total number of recommendations is shown as a bar plot. The bars are subdivided and colored according to the proportion of recommendations assigned to each CNS-WHO grade. HRD, homologous recombination deficiency. **c** Proportion of recommendations for combination therapies (either 1 targeted agent plus chemotherapy [light green] or 2 targeted agents [darker green]) and monotherapies (either one targeted agent [darkest green] or checkpoint inhibitor treatment [black]). **d** Proportion of recommendations based on NCT evidence levels (Methods). **e** Pie chart demonstrating the proportion of mutation types in the *PTEN* gene. **f** Sankey plot visualizing the result of immunohistological (IHC) PTEN analysis in tumors with *PTEN* mutations, demonstrating cases in which PTEN expression is retained (dark blue) by IHC and those in which PTEN is lost (dark green). IHC, Immunohistochemistry. NA not assessed. **g** Representative image of immunohistochemical slides showing (A) preserved PTEN expression and (B) loss of PTEN expression.

Treatment recommendations have been issued for 62 patients (71%) based on tumor molecular profiles and included a wide range of targeted and personalized therapeutic options (Fig. 3a). The rate of recommendations varied between CNS-WHO grades. 68% of CNS-WHO grade 4, 83% of CNS-WHO grade 3, 45% of CNS-WHO grade 2, and 91% of CNS-WHO grade 1 patients received treatment recommendations. These recommendations were classified based on the nationally established ‘NCT evidence levels’ (Methods)^21,22^. In patients with multiple therapy recommendations (*n* = 19), recommendations were prioritized considering these evidence levels. Therapies have been assigned to 9 biological processes and pathways, the majority related to cell cycle (*n* = 21), BRAF (*n* = 12), and mTOR signaling pathways (*n* = 10, Fig. 3b).

In total, we recommended targeted single-agent therapies for 41 patients and combinatory targeted drugs for 16 patients, while 8 patients received checkpoint inhibitor recommendations and 21 a combination of targeted therapies and alkylating chemotherapy (temozolomide, Fig. 3a,c). According to the national NCT evidence levels, the majority of recommendations were based on the strongest evidence level m1 (total of *n* = 47, 54%) derived from prospective studies or meta-analyses (m1A, *n* = 7), retrospective cohorts or case-control studies (m1B, *n* = 21), or from case reports and small case series in the same entity (m1C, *n* = 19), followed by evidence level m3 recommendations that are based on preclinical studies (*n* = 24, 28%, Fig. 3d). Only 17% of recommendations (*n* = 15) were supported solely by evidence derived from studies in different entities (m2, Fig. 3d).

In our cohort, *PTEN* mutations were the second most common alteration, occurring in 17 cases (Figs. 2 and 3e; Supplementary Data 4). All of these mutations were classified as pathogenic or likely pathogenic in either the OnkoKB or the Clinvar database, indicating loss of function of this tumor suppressor gene^23,24^. Yet, to validate PTEN loss on protein level before recommending biomarker-guided treatment with mTOR inhibitors, immunohistochemical analyses were performed in 12 cases, confirming PTEN protein loss in only 3 patients (25%, Fig. 3f, g). Notably, all genetic aberrations leading to PTEN loss were mutations in splice regions (Supplementary Data 4).

### Implementation of treatment recommendations

**Fig. 4:**
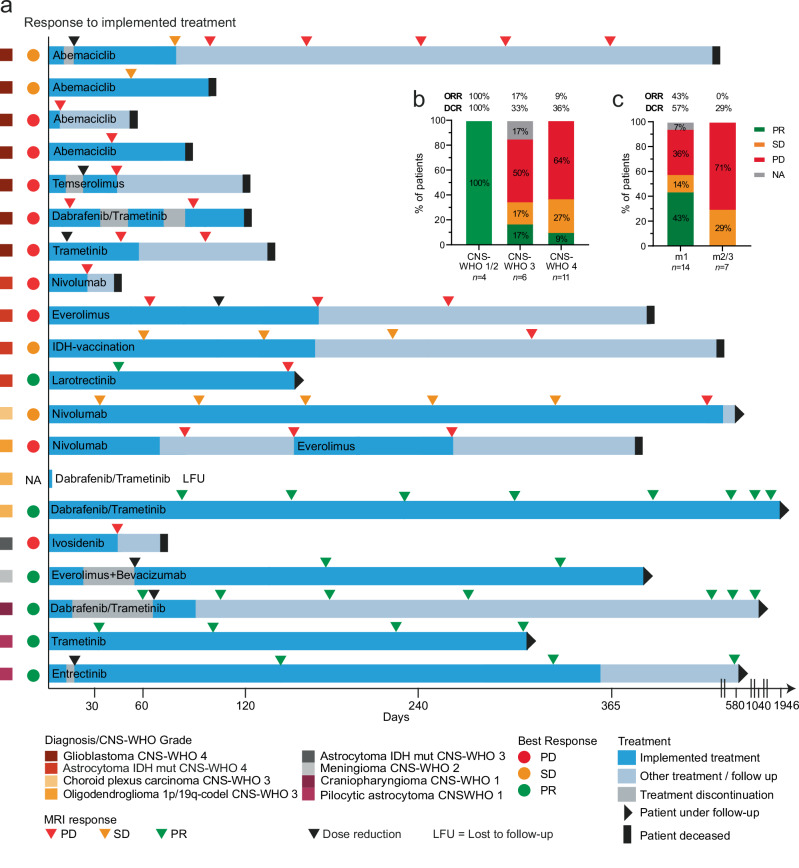
Response to implemented treatment. **a** Modified swimmer plot showing a patient-based treatment course and follow-up period. Time is given in days. The tumor entity is shown as a rectangle in a color code provided in the legend within the figure panel. The colored dot for each case represents the best response. Dark blue bars indicate the time of treatment with recommended therapy. Light blue bars indicate time without recommended treatment. Gray bars indicate discontinuation of the recommended treatment. Patients with a black triangle at the end of the bar are still being followed up. Patients with a black bar have died. MRIs during disease progression are shown with a triangle. The color is chosen according to the remission status as stated in the legend within the figure panel. Black triangles above the bars indicate the dose reduction of the recommended therapy. One patient was lost to follow-up immediately after starting treatment. No follow-up data is available for this patient. LFU loss to follow-up, wt wildtype, mut mutated, codel co-deleted, PD progressive disease, SD stable disease, PR partial remission. **b** Disease control rate (DCR) and overall response rate (ORR) achieved by recommended therapies among different CNS-WHO Grades. **c** DCR and ORR achieved by recommended therapies among different NCT evidence levels (m1 vs. m2/3).

Figure 4a and Supplementary Data 5 provide an overview of implemented biomarker-guided therapies and outcomes following the implementation of recommendations, ordered by CNS-WHO grades. Up to the data cut-off, a total of 20 patients were treated according to MTB-FR recommendations, resulting in a 32% implementation rate. This rate remained relatively stable across CNS-WHO grades, with implementation rates of 32% for CNS-WHO grade 4, 40% for CNS-WHO grade 3 and 2, and 27% for CNS-WHO grade 1 tumors. Reasons for non-implementation of therapies included rapid progression resulting in poor performance status or death (48%), LFU (14%), continuation of standard therapy with stable disease (31%), and patients refusing targeted treatment or health insurance did not cover the drug costs (5%) (Supplementary Data 1). In the 20 patients with implemented targeted therapies, a total of 21 therapies were administered including CDK4/6 inhibitors (*n* = 4), BRAF/MEK inhibitors (*n* = 4), mTOR inhibitors (*n* = 3), immunotherapies (*n* = 3), NTRK inhibitors (*n* = 2), MEK inhibitors (*n* = 2), IDH inhibitors (*n* = 1), IDH vaccination (*n* = 1), and a combination of VEGF and mTOR inhibition (*n* = 1) (Fig. 4a and Supplementary Data 5).

The median follow-up for patients who received recommended therapies was 181 days (range: 0–1946 days). At the time of the final analysis, 36% of these patients were alive and 16% continued to receive the recommended drug (Fig. 4a). They were heavily pretreated with a median of 3 prior treatment lines (Supplementary Data 5). The overall disease control rate (DCR) following implementation of recommended treatment was 50% and the overall response rate (ORR) was 30%, with 4 patients showing stable disease (SD) and 6 patients revealing a partial response (PR). Notably, DCR and ORR differed substantially between CNS-WHO grades and NCT evidence levels. While patients with CNS-WHO grade 1 or 2 tumors had DCRs and ORRs of 100%, DCRs and ORRs decreased to 33% / 36% and 17% / 9% in CNS-WHO grade 3 and 4 tumors, respectively (Fig. 4b). Similarly, patients receiving treatment based on m1 evidence levels showed a DCR of 57% and ORR of 43%, while the DCR for those with recommendations following m2/3 evidence levels was 29% and the ORR was 0% (Fig. 4c).

The median progression-free survival (PFS) of patients following implemented therapies was 104 days (range: 9–1946), with 15 events occurring during the follow-up period (Fig. 4a and Supplementary Data 5). 16 of 21 therapies (76%) met the criteria for PFS2/PFS1 ratio assessment and 20 therapies for Neuro-MCBS analysis, which are surrogate markers for the efficacy of biomarker-guided treatments (Methods)^25,26^. PFS2/PFS1 ratios ranged from 0.16 to 15.7. Among all evaluable patients, 5 (31%) showed a PFS2/PFS1 ratio of at least 1.3, and 8 patients (50%) had a ratio of ≥1.0 (Supplementary Data 5). Six out of 20 therapies (30%) achieved a Neuro-MCBS grade of 1, four therapies led to grade 2 (20%), and three therapies to grade 3 (15%) Neuro-MCBS. Seven therapies were assigned a score of 0 (35%), indicating no clinical benefit (Supplementary Data 5**)**. While the proportion of therapies leading to Neuro-MCBS grades 1 or 2 was highest in patients with CNS-WHO grade 1/2 tumors (100%), only 37% of patients with CNS-WHO grade 3/4 tumors were classified as Neuro-MCBS grade 1/2 (Supplementary Fig. 4a). Similarly, 61% of therapies based on NCT evidence level m1 resulted in Neuro-MCBS grades 1/2 and only 29% following m2/3 recommendations (Supplementary Fig. 4b).

Median overall survival (OS) after treatment implementation was 657 days (Supplementary Fig. 4c). In contrast, median OS for patients who did not receive recommended therapies was only 264 days, indicating a potential trend for a beneficial effect of biomarker-guided treatment in primary brain tumors (*p* = 0.086, Supplementary Fig. 4c). Key clinical characteristics did not differ between these two patient cohorts at MTB-FR enrollment (Supplementary Data 6).

One-third of patients experienced adverse events during recommended targeted treatment that required a dose reduction or temporary discontinuation of therapy (Fig. 4a and Supplementary Data 5). These included cytopenia (*n* = 2), exanthema (*n* = 2), infectious complications (*n* = 1), elevated liver enzymes (*n* = 1), hyperglycemia (*n* = 1), neurotoxicity (*n* = 1), musculoskeletal complaints (*n* = 1), and adrenal crisis (*n* = 1). Implemented treatment was permanently discontinued in two patients due to toxicity (Fig. 4a and Supplementary Data 5).

Finally, in a separate analysis, we compared key outcome parameters of our study with those from previous publications assessing targeted treatment options in brain cancer patients (Supplementary Data 7)^25,27–30^. Despite some differences in the study design, all relevant characteristics such as recommendation rate, rate of treatment implementation, and treatment responses were largely similar to these studies (Supplementary Data 7).

## Discussion

A strong discrepancy exists between the role of molecularly based characterization of primary brain cancers on the one hand and the availability of biomarker-guided treatment on the other hand. While the former has found its way into clinical routine and the WHO classification guidelines, the options for molecularly stratified therapies remain largely limited in CNS tumors. The anticipated approval of vorasidenib for *IDH*-mutated gliomas based on the results of the phase III INDIGO trial underscores the need for targeted treatment options in brain cancers^12^. Pioneering umbrella trials such as the N^2^M^2^ phase I/II or the INSIGhT phase II studies, which both explore the efficacy of molecularly informed therapeutic strategies in selected patients with glioblastoma without hypermethylation of the promotor of the O-6-methylguanine-DNA methyltransferase (MGMT), represent a significant stride towards this direction^31,32^. However, access to modern targeted therapies for most patients with brain cancers remains constrained, because a significant proportion of CNS tumor patients still does not qualify for most solid tumor umbrella trials, and due to the considerable lack of biomarker-informed trials for the majority of brain cancers other than glioblastoma.

MTBs at academic institutions combine the expertise of physicians, molecular pathologists, cancer biologists, geneticists, and bioinformaticians to offer personalized and biomarker-guided therapies after broad molecular testing for cancer patients without remaining standard-of-care treatment options^15–17,25^. We here presented clinical characteristics, results of comprehensive molecular profiling, and outcomes of 102 adult CNS tumor patients enrolled in the MTB-FR observational study between 2018 and 2023. The MTB-FR is one of four ‘Centers for Personalized Medicine (ZPM)’ in the state of Baden-Wuerttemberg, Germany. We demonstrated that molecular profiling of a broad variety of brain cancer entities is feasible in this real-world setting by successfully profiling 85% of patients initially presented at the MTB-FR. Aspects that limited the feasibility of our workflow were the availability of suitable tumor specimens and patient deaths before molecular diagnostics were completed. Furthermore, we showed that 71% of patients received one or more biomarker-informed treatment recommendations and that these recommendations were implemented in a substantial proportion of patients (32%). More than half of our recommendations (54%) were classified as NCT evidence level m1, demonstrating the MTB-FRs commitment to selecting therapies with the best possible anticipated efficacy. Yet, recommended therapies could not be implemented in a significant subset of patients (68%), mostly due to rapid progression resulting in poor performance status or death, indicating that brain cancer patients might benefit from earlier enrollment and initiation of the MTB-FR workflow. Another main reason for non-implementation was the unexpected prolonged response to standard treatment. In these patients, our recommended treatment options might be implemented in the future, which could further increase the implementation rate.

One major result of our study was that the implementation of personalized off-label therapies in this heavily pretreated patient cohort led to durable clinical response or disease control in a substantial proportion of patients. However, we noticed significant differences in treatment responses depending on tumor entities and whether recommended therapies were based on strong or weak evidence levels. While patients with lower-grade brain tumors (CNS-WHO grade 1/2) and those receiving therapies based on evidence level m1 showed the highest DCRs / ORRs and PFS2/PFS1 ratios, clinical benefit in patients with CNS-WHO grade 3 or 4 tumors and those with therapies in the m2/3 categories was substantially lower. In fact, only two patients with aggressive CNS-WHO grade 3 or 4 tumors had a partial response following targeted treatment with Larotrectinib and Dabrafenib/Trametinib, both implemented based on evidence level m1 after detection of an *NTRK* fusion and a *BRAF* V600E mutation. These findings generally suggest that patients in this clinical context tend to derive the greatest benefit from therapies with documented efficacy within the same cancer entities in early trials or case reports, as well as from established treatment modalities in other cancer types such as NTRK inhibitors or BRAF/MEK inhibitors^33,34^. This notion reflects similar observations of previous studies evaluating targeted therapies in primary brain tumors^25,27,28^. In contrast, targeted treatment approaches that rely on evidence from preclinical studies were clearly less effective, indicating the need for refining and optimizing the selection of patients for these drugs and the design of future innovative biomarker-informed clinical trials.

Key characteristics of our study were largely comparable to other published studies in the field, both in terms of recommendation rates and responses to recommended therapies, despite some differences in brain cancer entities considered for these analyses^25,27–29^. For example, our implementation rate of 32% and the PFS 2/1 ratio of 31% were largely similar to those reported in the other brain cancer studies^5,17,25,27–30,35^. Furthermore, our results were also in line with publications that explored molecularly stratified therapies in other solid cancers^5,17^. For example, implementation rates of biomarker-guided therapies were 31.8% and 23.6% and PFS 2/1 ratios above 1.3 were reported for 35.7% and 33% of patients in large studies profiling genetic alterations in rare and advanced solid tumors, mirroring the results of our work^5,17^.

A notable result of our study was the substantial number of identified *PTEN* mutations that were classified as pathogenic or likely pathogenic in different databases. However, only a subset of these cases showed a PTEN loss by immunohistochemistry, indicating no effect on the mTOR signaling pathway. To further explore the activation of the mTOR signaling pathway, other studies incorporated immunohistochemical analyses for phospho-mTOR or phosphor-S6^25,32^. Up to this date, we have not included these biomarkers in our portfolio at the MTB-FR, potentially missing patients who are eligible for mTOR inhibition.

Our work harbors several limitations and various hurdles remain to overcome. First, an inherent limitation is the relatively small sample size and single-center nature of our study and the heterogeneity of patient cohorts, tumor entities, and applied therapies, which hamper the interpretation of clinical outcomes. We have used measures such as the PFS2/PFS1 ratio and Neuro-MCBS to estimate the efficacy of targeted treatment strategies. Yet, although the PFS2/PFS1 ratio is widely used in this context, different thresholds have been applied, introducing some uncertainties around its interpretability^17,25,35^. The Neuro-MCBS has been developed recently, particularly for neuro-oncological patients; yet, it requires further validation in prospective clinical trials to demonstrate its value^25^. Furthermore, due to technical advances over time, the landscape of available sequencing methods in this real-world setting has changed during our observational study, introducing some heterogeneity of technologies applied for tumor genetic profiling. The genomic coverage has increased over the years, introducing the risk that earlier panels might have missed some therapeutically relevant targets. However, since the vast majority of targetable alterations in primary brain tumors can be captured by rather small panels, the technical progress in sequencing technologies probably had only a minor impact on the recommendation rates. Finally, log-rank analyses showing survival advantages of patients with implemented therapies compared to those who did not receive targeted treatment must be interpreted with caution, because the general condition of the latter cohort was likely more reduced at the time of therapy recommendation.

Collectively, our study provides real-world evidence for the efficacy of molecularly guided treatment approaches in a subset of brain cancer patients, highlighting the need for biomarker-stratified trials in these entities and informing future research projects to further refine individualized therapeutic strategies in these patients.

## Methods

### Patient cohort and study design

All patients included in this analysis were diagnosed with primary brain tumors and enrolled in the MTB-FR observational study (DRKS00025847) from September 2018 to October 2023 for comprehensive molecular profiling and real-world biomarker-informed stratification of therapies. Data cutoff was October 31st, 2023 for enrollment in the MTB-FR and June 1st, 2024 for outcome assessment. The MTB-FR is represented by a single-center multidisciplinary team of physicians from more than 16 different departments, experts from molecular pathology, molecular biology, and medical bioinformatics^36^. Our analysis included adults (aged 18 years or older) with primary brain cancers treated at the University Medical Center Freiburg, Germany, who were registered at the MTB-FR by the treating physician for the observational study due to a lack of standard treatment options. The MTB-FR observational study was approved by the Ethics Committee of the University of Freiburg Medical Faculty and conducted in accordance with the Declaration of Helsinki (ethic vote number 369/19). All patients provided written informed consent. Recommendations for entity-specific molecular analyses and treatment recommendations follow standard operation procedures as previously described and depicted in Fig. 1a^36^. In brief, patient cases are initially presented at the MTB-FR by the treating physician, followed by comprehensive molecular profiling of their tumors. Then, potential treatment recommendations are given based on these molecular analyses following interdisciplinary discussions and consensus, classified according to the nationally established NCT evidence levels^21,22,36^: m1, evidence in the same entity; m2, evidence in a different entity; m3, preclinical evidence; m4, biological rationale; m1 and m2 have three suffixes: A, evidence from prospective trial or meta-analysis; B, evidence from retrospective cohorts or case-control study; C, case study or single unusual responder.

### Molecular profiling

The most suitable tumor material was selected for molecular profiling by the MTB-FR at the initial presentation (Fig. 1a). Either formalin-fixed paraffin-embedded (FFPE) tissue or fresh frozen tumor samples were profiled. After morphological microdissection of the tumor cells, automated tumor DNA and RNA extraction were performed (Maxwell® RSC DNA and RNA FFPE Kit/s; Maxwell® RSC48, Promega). Extracted DNA and RNA were then checked for quantity (Qubit, Thermo Fisher Scientific and TapeStation, Agilent) and quality (DIN/RIN, TapeStation, Agilent). DNA for germline analyses was extracted from plasma-depleted whole blood (PDWB) using the QIAamp DNA Mini kit (QIAGEN) according to the manufacturer’s instructions.

Diagnostic immunohistochemistry (PTEN, PD-L1, Somatostatin Receptor, HER2, phosphorylated ERK) and targeted NGS using the TSO500 panel (*n* = 57, including DNA analyses for 523 genes with SNVs, TMB, MSI, CNV as well as RNA analyses for 55 genes with RNA-Fusions and RNA splice variants; Illumina), the DNA-based TruSeq Amplicon Cancer Panel (*n* = 2, including 48 genes; Illumina), the DNA-based custom NGS-Panel of the national network of genomic medicine in lung cancer (nNGM-Panel; *n* = 6, including “Hotspot” regions/exons of 19, respective 27 genes in V1.0 and V2.1, Qiagen) and/or a large RNA-Fusion panel (*n* = 2, 507 genes; Illumina) were performed by the Institute of Surgical Pathology according to accredited diagnostic workflows.

Libraries of tumor and germline DNA for WES (*n* = 12) were prepared using SureSelect XT and SureSelect V5 + UTR or SureSelect XT and SureSelect V6 + UTR target enrichment kits, all provided by Agilent (Santa Clara, CA, USA). Sequencing was performed on the Illumina sequencing platform (HiSeq 4000 / NovaSeq 6000). After sequencing, quality control and trimming with FASTQC and Trimmomatic was performed. Further, we used Mutect2 and VarScan2 for variant calling, followed by false positive filtering according to GATK best practices^37–41^. Tumor somatic variants were distinguished from germline variants by comparing tumor sequencing results with those from the matched germline. Only non-silent single nucleotide variants (SNVs) and small insertions/deletions (InDels) with a variant allele frequency (VAF) greater than 5% and with a population frequency of less than 0.1% (MAF, minor allele frequency) in the Genome Aggregation Database (gnomAD) were reported^42^. Cancer genes were annotated according to ClinVar, InterVar, OncoKB, and cancer hotspots, and functionally annotated according to the dbNSFP database that contains 38 predictions and eight conservation scores^23,43–48^.

RNA sequencing was performed separately from the TSO500 analysis for the identification of structural rearrangements in 14% of cases. RNA libraries were prepared using SmarTer Ultra Low Input RNA v4 (Takara Bio Group) and sequenced on Illumina sequencing platforms as reported above. After RNA sequencing, fusions were inferred with FusionCatcher and arriba^49^. The results were visualized using the R framework and the Bioconductor package maftools^50,51^ and in a customized^52–54^ version of cBioPortal.

Finally, eight patients underwent external tumor DNA sequencing using the NPHD2015A panel, which targets 130 genes and was specifically designed for glioblastoma^55,56^.

### Clinical response criteria and outcomes

Clinical data and pathological reports for all patients were retrospectively assessed and reviewed for this analysis. Tumor classification was made according to the 2021 WHO classification. The radiological response was determined based on the Response Assessment in Neuro-Oncology (RANO) criteria for high-grade/low-grade glioma^57,58^. Progression-free survival (PFS) was defined as the time from the start of recommended treatment to the first radiological progression or death from any cause or follow-up. Overall survival (OS) was calculated from the start of recommended treatment to the date of death from any cause or last follow-up. Patients were excluded from the outcome analysis if there was a loss of follow-up before treatment recommendations were made (*n* = 5).

To specifically evaluate response to the recommended targeted treatment in comparison to previous standard therapies, the PFS 2/1 ratio was calculated as follows: PFS following the recommended therapy by the MTB-FR (PFS 2) / PFS following the prior line of treatment (PFS 1). Yet, we considered only cases for the analysis of PFS 2/1 ratios if the follow-up after implementation of recommended treatments was at least as long as PFS following the previous treatment line. Furthermore, outcomes of recommended therapies were also classified using the Neuro-MCBS, as described by Renovanz et al.^25^. DCR described the proportion of patients who achieved complete response (CR), PR, or SD in response to the recommended therapeutic intervention. ORR was defined as achieving CR or PR in response to the recommended treatment.

### Statistical analyses

Continuous variables were presented using median and range. Time-to-event variables were visualized using the Kaplan-Meier method and the log-rank test was used to evaluate survival differences.

### Supplementary information

Data_Supplement

Supplementary_Data

## Data Availability

Pseudonymized clinical and demographic data for cases considered in this study, as well as tumor mutational data and other relevant data, are provided in the Supplementary Data. Owing to restrictions related to the dissemination of germline sequence information included in the informed consent forms used to enroll study subjects in the MTB-FR observational study, we are unable to provide access to raw sequencing data. Reasonable requests for additional data will be reviewed by the authors to determine whether they can be fulfilled in accordance with these privacy restrictions.

## Code availability

The analyses were conducted using existing software and tools, which are detailed in the Methods section. Should there be any inquiries regarding the computational methods employed, please contact the corresponding author for further information.
