# Supplementary material for: Comprehensive genetic profiling and molecularly guided treatment for patients with primary CNS tumors
Source: NPJ Precis Oncol. 2024 Aug 14;8:180. doi: 10.1038/s41698-024-00674-y (PMC11324882; doi:10.1038/s41698-024-00674-y)
Supplement: Supplementary file 1 — Data_Supplement [file 41698_2024_674_MOESM1_ESM.pdf]

Data Supplement for

Comprehensive genetic profiling and molecularly guided treatment for patients with primary CNS tumors

Julia C. Kuehn, Patrick Metzger, Nicolas Neidert, Uta Matysiak, Linda Gräßel, Ulrike Philipp, Sabine Bleul, Thomas Pauli, Julia Falkenstein, Henriette Bertemes, Stepan Cysar, Maria Elena Hess, Anna Verena Frey, Jesús Duque-Afonso, Elisabeth Schorb, Marcia Machein, Jürgen Beck, Oliver Schnell, Nikolas von Bubnoff, Anna L. Illert, Christoph Peters, Tilman Brummer, Marco Prinz, Cornelius Miething, Heiko Becker, Silke Lassmann, Martin Werner, Melanie Börries, Justus Duyster, Dieter H. Heiland, Roman Sankowski, Florian Scherer^

^ Corresponding author. Email: florian.scherer@uniklinik-freiburg.de

<u>**Table of contents**</u>

Supplementary Data – attached separately as Excel file

Supplementary Data 1. Overview of patients in which treatment recommendations were not implemented.

Supplementary Data 2. Samples used for molecular profiling.

Supplementary Data 3. SNVs detected in 87 brain tumor samples.

Supplementary Data 4. List of PTEN mutations and respective results of PTEN immunohistochemistry.

Supplementary Data 5. Detailed list of implemented treatment recommendations and clinical outcomes.

Supplementary Data 6. Characteristics of patients with implemented treatment recommendations in comparison to patients without implementation of treatment recommendations.

Supplementary Data 7. Comparison with other studies.

Supplemental Figures

Supplementary Figure 1

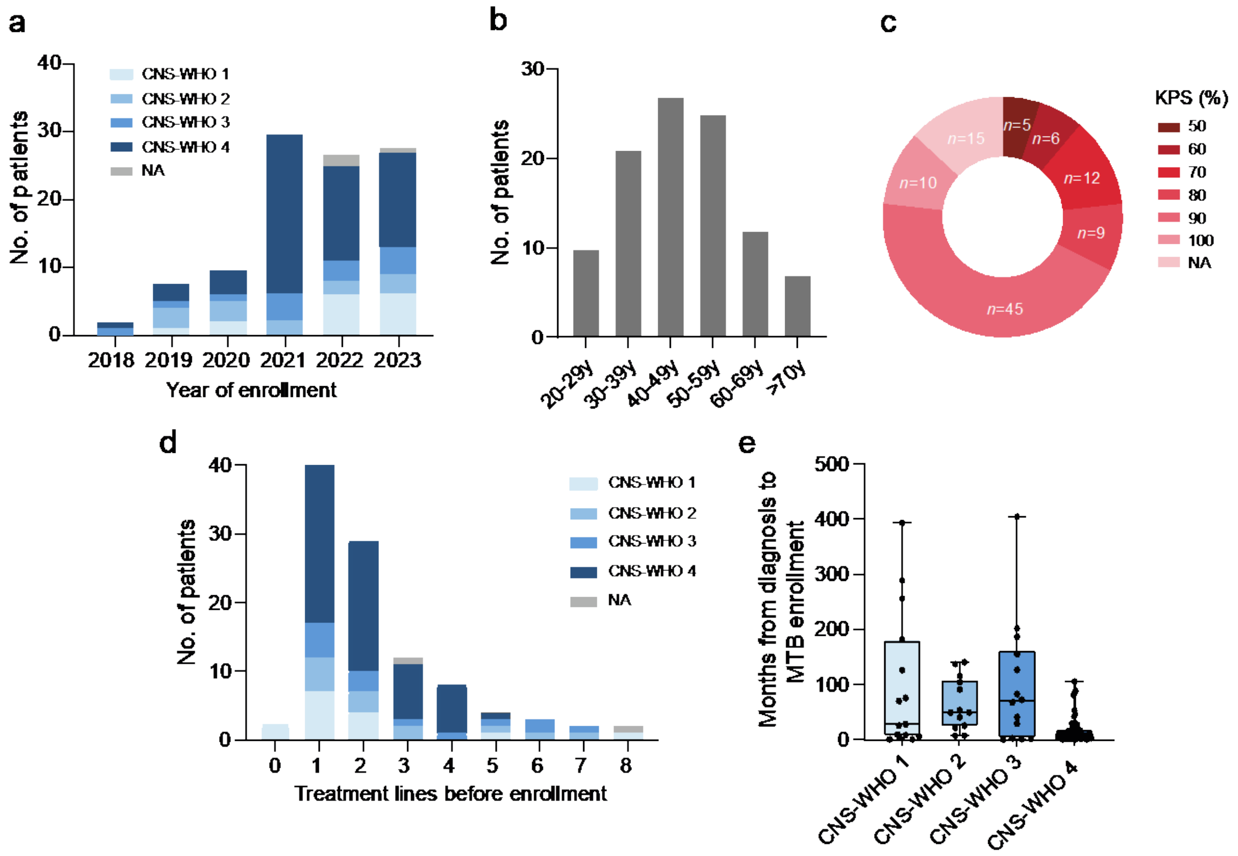

Supplementary Figure 1: Characteristics of the patient cohort. (a) Bar graph depicting the number of primary brain tumor patients enrolled in the MTB-FR observational study per year from 2018 to 2023, ordered by CNS-WHO grades. No., number. **(b)** Age distribution of primary brain tumor patients enrolled in the MTB-FR observational study. No., number; y, years. **(c)** Pie chart showing the Karnofsky performance status (KPS) of enrolled patients. **(d)** Number of treatment lines before enrollment in the MTB-FR observational study, ordered by CNS-WHO grades. No., number. **(e)** Median time from initial diagnosis to enrollment in the MTB-FR observational

study in months for different CNS-WHO grades. Line, median; Box, 25-75th percentile; Error bars, range.

Supplementary Figure 2

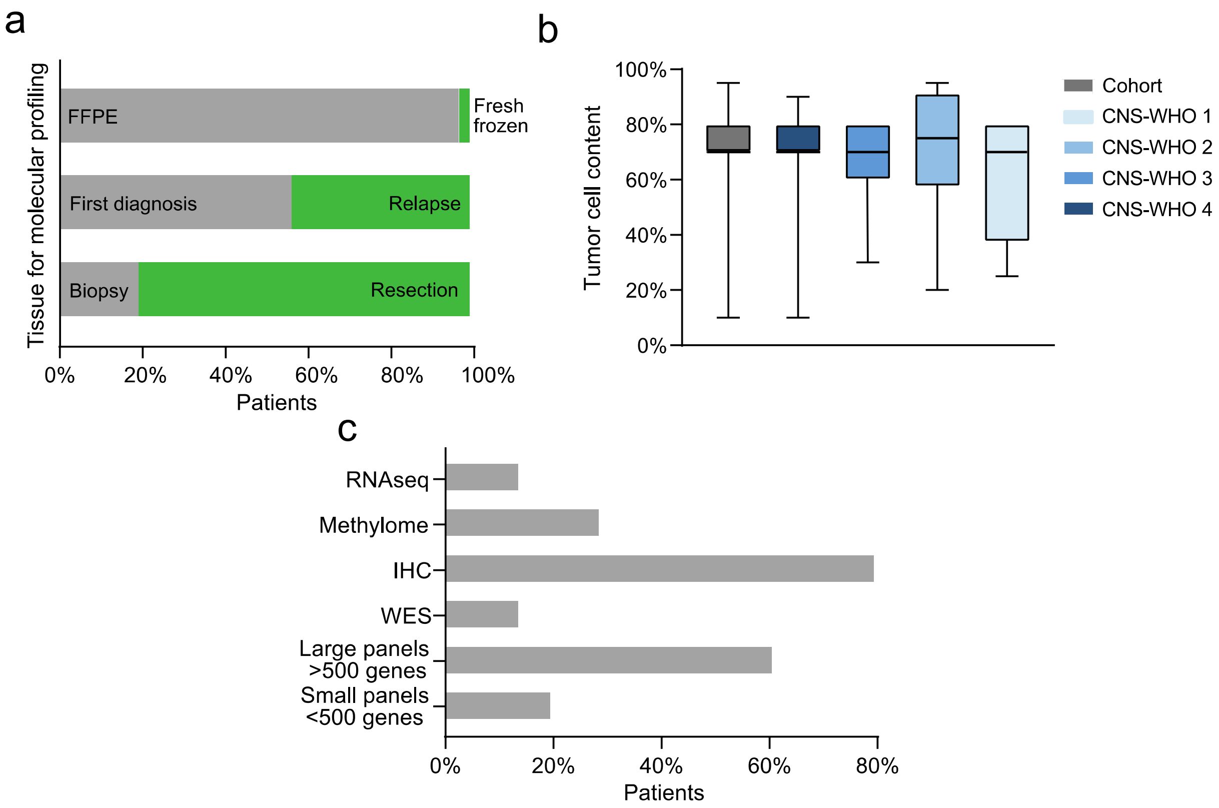

Supplementary Figure 2: Tumor molecular profiling. (a) Characteristics of tumor tissue used for molecular profiling. Upper bar: Proportion of FFPE vs fresh frozen tissue. Middle bar: Proportion of tissue obtained from initial diagnosis vs tissue from a relapse time point. Lower bar: Proportion of tissue obtained by a stereotactic biopsy vs brain tumor resection. FFPE, formalin-fixed paraffin-embedded. **(b)** Distribution of tumor cell content in tumor specimens. Grey bar: entire cohort; dark to light blue bars: different CNS-WHO grades. Line, median; Box, 25-75th percentile; Error bars, range. **(c)** Methods used for molecular profiling. Grey bars showing the proportion of samples being analyzed with the respective method. RNA sequencing (RNAseq); Immunohistochemistry (IHC); Whole exome sequencing (WES); Targeted next-generation sequencing using large panels (>500 genes); Targeted next-generation sequencing using small panels (<500 genes).

Supplementary Figure 3

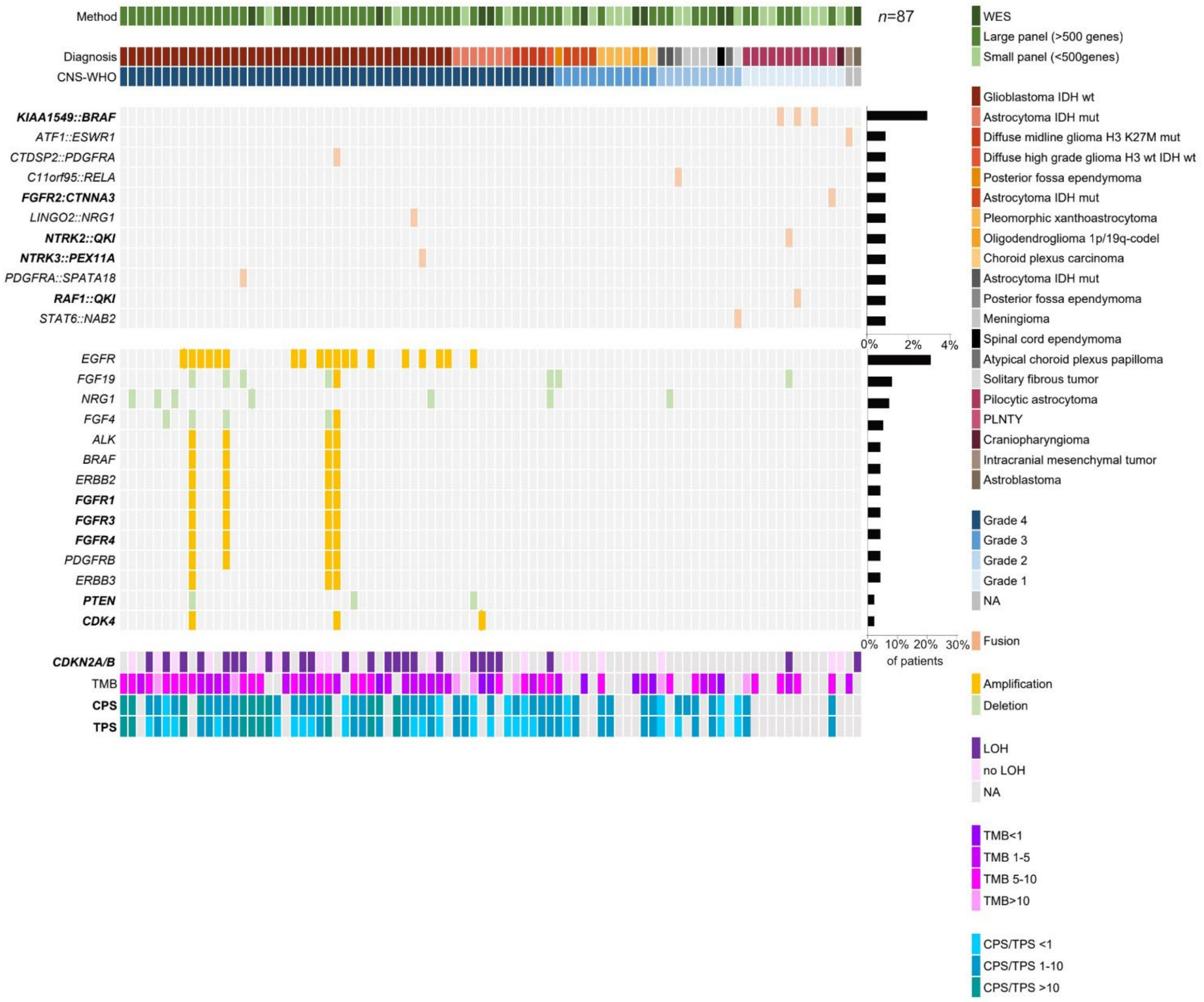

Supplementary Figure 3: Fusions and copy number variants (CNVs) detected in tumor specimens. Case-level fusion/CNV/other biomarker profiles of 87 primary brain tumors. Each column represents a tumor sample, each row represents a gene/fusion/biomarker. Amplifications/Deletions occurring in at least 3 cases are shown. All detected fusions are shown. The percentage of patients carrying an alteration is shown as a bar graph on the right. Sequencing method, diagnosis as well as WHO grades are depicted in the first three rows. Bold gene/biomarker names indicate alterations resulting

in therapy recommendations. TPS is given in percent. WES, Whole exome sequencing; wt, wildtype; mut, mutated; codel, co-deleted; PLNTY, polymorphous low-grade neuroepithelial tumor of the young; LOH, loss of heterozygosity; TMB, tumor mutational burden; CPS, Combined Positive Score; TPS, Tumor Proportion Score.

Supplementary Figure 4

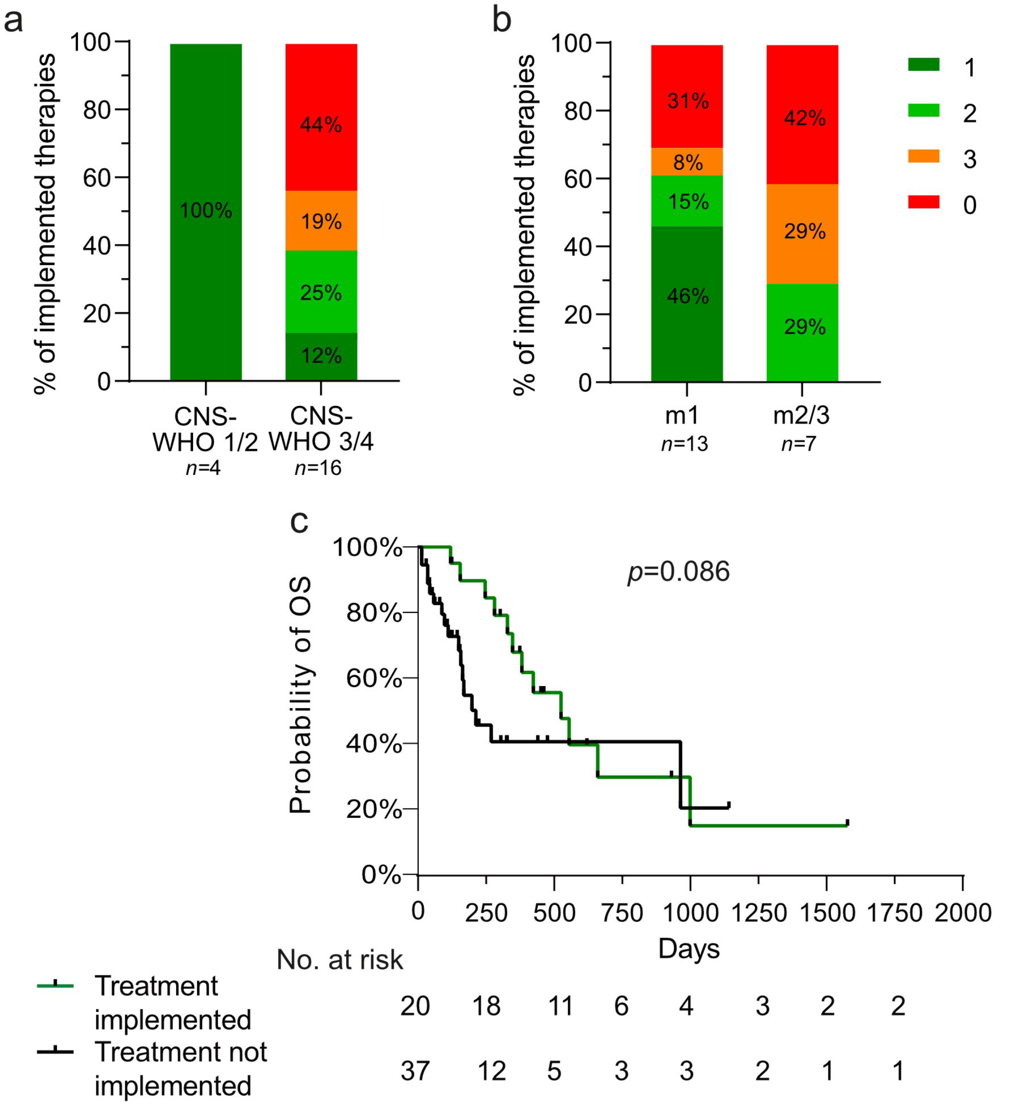

Supplementary Figure 4: Clinical outcomes following recommended therapies. (a) Bar plot showing Neuro-MCBS grades 0-3 in patients with either CNS-WHO grade 1/2 or CNS-WHO grade 3/4 tumors. **(b)** Bar plot showing Neuro-MCBS grades 0-3 in patients with treatment implemented according either NCT evidence level m1 or m2/3. **(c)** Kaplan-Maier curve comparing overall survival of patients with implemented treatment

recommendations (green) and patients in which recommended treatment was not implemented. OS, overall survival; No., number.
